# Sphingolipids of Asteroidea and Holothuroidea: Structures and Biological Activities

**DOI:** 10.3390/md19060330

**Published:** 2021-06-08

**Authors:** Timofey V. Malyarenko, Alla A. Kicha, Valentin A. Stonik, Natalia V. Ivanchina

**Affiliations:** 1 G.B. Elyakov Pacific Institute of Bioorganic Chemistry, Far Eastern Branch of the Russian Academy of Sciences, Pr. 100-let Vladivostoku 159, 690022 Vladivostok, Russia; kicha@piboc.dvo.ru (A.A.K.); stonik@piboc.dvo.ru (V.A.S.); 2 Department of Bioorganic Chemistry and Biotechnology, School of Natural Sciences, Far Eastern Federal University, Sukhanova Str. 8, 690000 Vladivostok, Russia

**Keywords:** sphingolipids, ceramides, cerebrosides, gangliosides, sialic acid, Asteroidea, Holothuroidea, biological activity, neuritogenic activity

## Abstract

Sphingolipids are complex lipids widespread in nature as structural components of biomembranes. Commonly, the sphingolipids of marine organisms differ from those of terrestrial animals and plants. The gangliosides are the most complex sphingolipids characteristic of vertebrates that have been found in only the Echinodermata (echinoderms) phylum of invertebrates. Sphingolipids of the representatives of the Asteroidea and Holothuroidea classes are the most studied among all echinoderms. In this review, we have summarized the data on sphingolipids of these two classes of marine invertebrates over the past two decades. Recently established structures, properties, and peculiarities of biogenesis of ceramides, cerebrosides, and gangliosides from starfishes and holothurians are discussed. The purpose of this review is to provide the most complete information on the chemical structures, structural features, and biological activities of sphingolipids of the Asteroidea and Holothuroidea classes.

## 1. Introduction

Being the second-largest clade in a superphylum Deuterostomia after chordates, Echinodermata (echinoderms) is a phylum of exclusively marine invertebrates, inhabiting all the oceans in all the depths. These animals are characterized by radial symmetry, a particular water vascular system, and calcareous particles (ossicles) embedded in the dermis of their body walls. In some habitats, echinoderms are the dominant species in marine communities. There are five living classes of Echinodermata: Holothuroidea (sea cucumbers), Asteroidea (starfish), Ophiuroidea (brittle stars), Echinoidea (sea urchins), and Crinoidea (sea lilies and feather stars). These invertebrates present a rich source of diverse low molecular biologically active metabolites, including triterpene glycosides, polar steroids, and their glycosides, peptides, fatty acids, carotenoids, quinoid pigments, and different lipids, including sphingolipids. Our group is carrying out long-term studies on natural products from echinoderms [1,2,3,4,5,6], but sphingolipids from these invertebrates [7] so far were not in our main spotlight. However, our recent metabolomic studies on secondary metabolites from echinoderms, showing their extremal diversity [8,9,10,11,12,13], and successful attempts of application of some compounds as chemotaxonomic markers required the examination of perspectives of similar use of sphingolipids.

Sphingolipids, a group of heterogeneous lipids known as constituents of the plant, fungal, and animal cellular membranes, play a fundamental role in important phenomena such as cell-cell recognition and antigenic specificity [14,15]. Sphingolipids include ceramides, the hydrophobic molecules, involving a long-chain base (LCB) and an amide-linked fatty acid residue (FAR) and their glycoconjugated derivatives. Glycosylated ceramides are named cerebrosides, except for the corresponding oligoglycosides with carbohydrate chains, comprising one, two, three, or more sialic acid residues, which are known as gangliosides [16]. Sphingolipids were isolated from a number of biological sources, including marine invertebrates such as sea anemones [17], sponges [18,19,20], octocorals [21], ascidians [22], and representatives of other taxa. Various biological activities of ceramides, cerebrosides, and gangliosides, including plant growth stimulatory action [23], anti-inflammatory effects [24], the improving of the barrier function of the skin [25], cancer-protective action [26], proangiogenic action [27] have been reported.

In their majority, reviews about sphingolipids from marine organisms [28], including those concerning the corresponding natural products from echinoderms, were published from 12 to 20 years ago [29,30,31]. The present review includes data concerning chemical structures of sphingolipids from two classes of the phylum Echinodermata and their biological activities and covers the literature from 2000 to March 2021. We have focused our attention on the structures of these compounds, modern methods of analyses of complicated fractions of these lipids, and their bioactivities. Current problems of these studies are also discussed.

## 2. Ceramides

**Figure 1:**
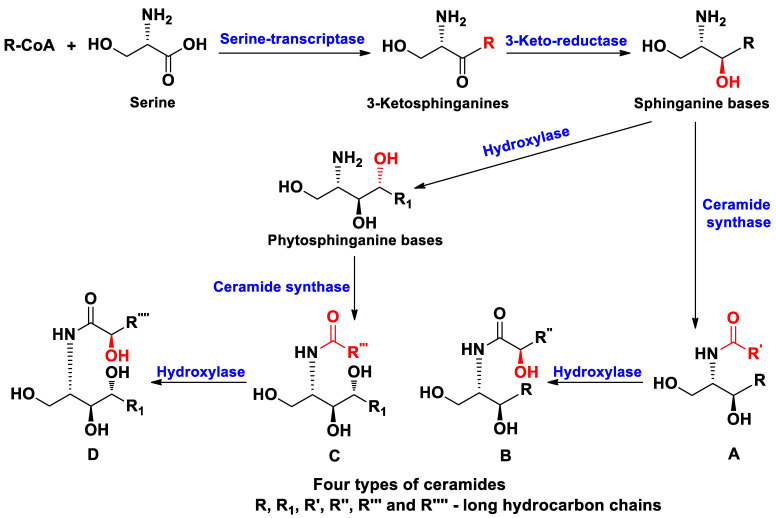
Scheme of biosynthesis and structures of main types of ceramides in echinoderms.

Ceramides are biosynthesized at the reaction of S-acyl-coenzyme A (usually C_16_-CoA) with serine, catalyzed by serine palmitoyl transcriptase or related enzymes, followed by reduction of carbonyl group by ketosphinganine reductase and the N-acylation by ceramide synthase. Surprisingly, hydroxylation of long-chain bases (LCBs) that leads to so-called phytosphinganine derivatives, takes a place in plants and in many echinoderms. When hydroxylases act on fatty acid residues (FARs) in these invertebrates, an additional hydroxyl is introduced also into α–position of FARs [32]. As result, four main types of ceramides are known from different organisms including echinoderms, namely, A—containing sphinganine bases and nonhydroxylated fatty acid residues, B—consisting of sphinganine bases and α–hydroxylated fatty acids, C—containing phytosphinganine bases and nonhydroxylated fatty acids, and D—consisting of phytosphinganine bases and α–hydroxylated fatty acids (Figure 1). Both bases and fatty acids moieties in this type of natural products may contain normal chains, as well as those with *iso*- and/or *anteiso*-branching. Therefore, ceramides have great structural variety.

### Class Asteroidea

**Figure 2:**
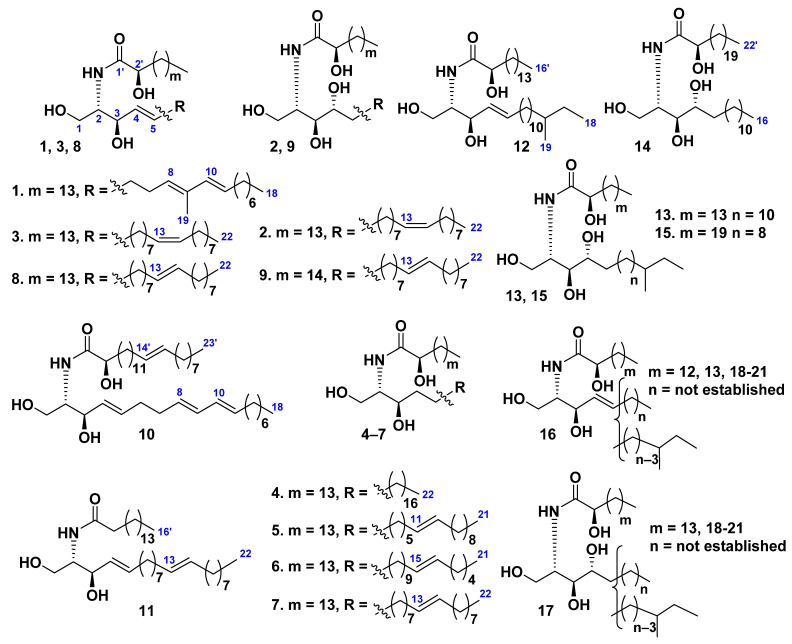
Ceramides from the starfish *Distolasterias nipon* and *Luidia maculata*.

Three ceramides **1**–**3** were isolated from the starfish *Distolasterias nipon* collected off the coast of the East Sea, Republic of Korea [33]. Structures of **1**–**3** were established by spectroscopic techniques and chemical transformations as (2*S*,3*R*,4*E*,8*E*,10*E*)-2-[(2*R*)-2-hydroxyhexadecanoylamino]-9-methyl-4,8,10-octadecatriene-1,3-diol (**1**), (2*S*,3*S*,4*R*,7*Z*)-2-[(2*R*)-2-hydroxyhexadecanoylamino]-7-docosene-1,3,4-triol (**2**), and (2*S*,3*R*,4*E*,7*E*)-2-[(2*R*)-2-hydroxyhexadecanoylamino]-7-docosene-1,3,4-triol (**4**) (Figure 2). Later ceramides **2** and **3** (iteratively) and **4**–**11** (additionally) were extracted from the same species of starfish and purified by silica gel column chromatography and reversed-phase high-performance liquid chromatography [34]. The high-energy collision-induced dissociation (CID) spectra of ceramides with various structures, differing from each other in the number and positions of double bonds on both the *N*-acyl and sphingoid chains as well as in the presence of hydroxy groups or a double bond at the C-4 position of the sphingoid chains as well as an additional α-hydroxy group in *N*-acyl chains, were established. The CID mass spectrum of the monosodiated ion [M + Na]^+^ of each ceramide molecular species provided structural data concerning fatty acyl chains and sphingoid long-chain bases. This technique allowed determining complete structures of ceramides and cerebrosides in a mixture of sphingoid lipids and showed great potential for analysis of other sphingolipids isolated from various biological sources [34].

A sphingosine-type ceramide LMCer-1-1 (**12**) and three phytosphingosine-type ceramides, LMCer-2-1 (**13**), LMCer-2-6 (**14**), and LMCer-2-7 (**15**), were isolated from the ceramide molecular species LMCer-1 (**16**) and LMCer-2 (**17**), obtained from the chloroform–methanol extract of the whole bodies of *Luidia maculata* [35]. The structures of ceramides **12**–**15** were determined on the basis of spectroscopic and chemical evidence as (2*S*,3*R*,4*E*)-2-[(2*R*)-2-hydroxyhexadecanoylamino]-16-methyl-4-octadecene-1,3-diol (**12**), (2*S*,3*S*,4*R*)-2-[(2*R*)-2-hydroxyhexadecanoylamino]-16-methyl-octadecane-1,3,4-triol (**13**), (2*S*,3*S*,4*R*)-2-[(2*R*)-2-hydroxydocosanoylamino]-hexadecane-1,3,4-triol (**14**), and (2*S*,3*S*,4*R*)-2-[(2*R*)-2-hydroxydocosanoylamino]-14-methyl-hexadecane-1,3,4-triol (**15**) (Figure 2).

The phytosphingosine-type ceramide asteriaceramide A was isolated from the whole bodies of the Northern Pacific starfish *Asterias amurensis* [23]. The structure of this compound was determined as identical to compound **2**. Asteriaceramide A (**2**) showed a stimulatory activity toward root growth of *Brassica campestris*. The plant growth activity of the ceramide was reported for the first time.

Since 2000, there were no data on the isolation, structure elucidation, and determination of biological activities of sea cucumber ceramides.

Thus, representatives of all the above-mentioned structural groups of ceramides (Figure 1A–D) were found from starfish. The structural diversity of these metabolites is connected with the presence of many variants of both sphingoid and fatty acid moieties. It should be noted that generally ceramides from starfish were studied worse than other groups of sphingolipids. Perhaps this is due to the difficulty of isolation of individual ceramides or their molecular species.

## 3. Cerebrosides

Cerebrosides are glycosylceramides that contain glucose, galactose, or other monosaccharide residues in their carbohydrate moieties. These compounds are synthesized by enzymes: UDP-glucose:ceramide β-d-glucosyl-transferase, UDP-galactose:ceramide β-d-galactose-transferase, and other glycosyl-transferases [32]. Cerebrosides can be divided into three classes: monoglycosides, biglycosides (mainly lactosides), and oligoglycosides. This class of complex lipids can contain an aminosugar residue (globosides) in their carbohydrate moieties or be sulfated at a terminal monosaccharide residue [30]. Cerebrosides, such as ceramides, are part of the plasmatic membranes of cells and perform a number of important biological functions: they take part in the formation of new membranes, such as phospholipids, sterols, and cellular membrane proteins, and also participate in the transmission of cellular signals [14]. Moreover, the cerebrosides in cellular membranes act as cell surface antigens and receptors. Interest in sphingolipids and their derivatives mainly is associated with their high biological significance. Some studies have shown that sphingolipids can inhibit the growth of microalgae, fungi, and bacteria. The presumptive mechanism of this action is associated with the ability of this type of compound to perforate cell membrane, in addition, in the presence of sphingolipids, the ability of bacterial cells to adhere is reduced [36,37,38]. The ability of sphingolipids to stimulate plant growth [23], demonstrate an anti-inflammatory effect [24], and improve the barrier function of the skin [25] has also been shown.

An even larger variety of cerebrosides containing one or more monosaccharide residues, in comparison with ceramides, was isolated from starfish and sea cucumbers.

### 3.1. Class Asteroidea

**Figure 3:**
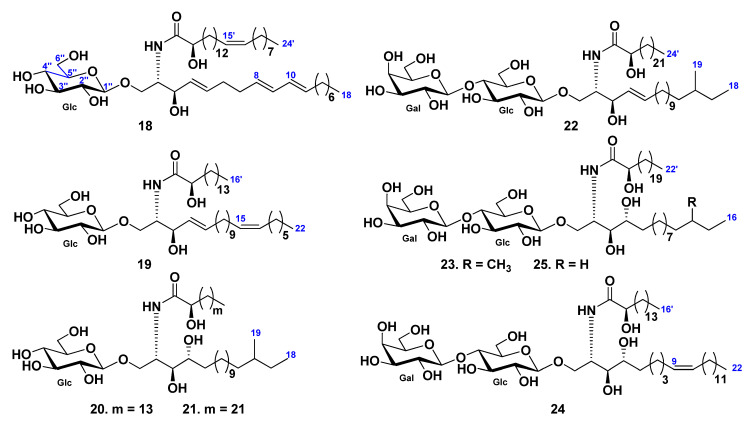
Cerebrosides from the starfish *Allostichaster inaequalis* and *Luidia maculata*.

From the chloroform–methanol–water extract of gonads and body walls of the Patagonian starfish *Allostichaster inaequalis,* glucosylceramides were isolated [39], along with the previously known phalluside-1 and two glucosylceramides earlier isolated from the starfish *Cosmasterias lurida* [40]. Compounds were described as (2*S*,3*R*,4*E*,8*E*,10*E*)-1-*O*-(β-d-glucopyranosyl)-2-[(2*R*)-2-hydroxy-15-tetracosenoylamino]-4,8,10-octadecatrien-3-ol (**18**) and (2*S*,3*R*,4*E*,15*Z*)-1-*O*-(β-d-glucopyranosyl)-2-[(2*R*)-2-hydroxyhexadecanoylamino]-4,15-docosadien-3-ol (**19**) using spectroscopic and chemical methods (Figure 3).

Glucosylcerebrosides, luidiacerebrosides A (**20**) and B (**21**), were isolated from the cerebroside fraction obtained from the extract of the starfish *Luidia maculata* using HPLC [41]. The structures of cerebrosides were determined as 1-*O*-β-d-glucopyranoside of (2*S*,3*S*,4*R*)-2-[(2*R*)-2-hydroxyhexadecanoylamino]-16-methyl-octadecane-1,3,4-triol (**20**) and (2*S*,3*S*,4*R*)-2-[(2*R*)-2-hydroxytetracosanoylamino]-16-methyl-octadecane-1,3,4-triol (**21**), respectively [41]. In continuation of the studies on sphingolipids from the same starfish four cerebrosides, luidialactosides A–D (**22**–**25**), were isolated from its the water-insoluble lipid fraction [42]. They were proved to contain lactosyl carbohydrate chains attached to C-1 of 2-[(2*R*)-2-hydroxytetracosanoylamino]-16-methyl-4-octadecene-1,3-diol (**22**), 2-[(2*R*)-2-hydroxydocosanoylamino]-14-methyl-1,3,4-hexadecanetriol (**23**), 2-[(2*R*)-2-hydroxyhexadecanoylamino]-9-docosene-1,3,4-triol (**24**), and 2-[(2*R*)-2-hydroxydocosanoylamino]-1,3,4-hexadecanetriol (**25**) (Figure 3).

**Figure 4:**
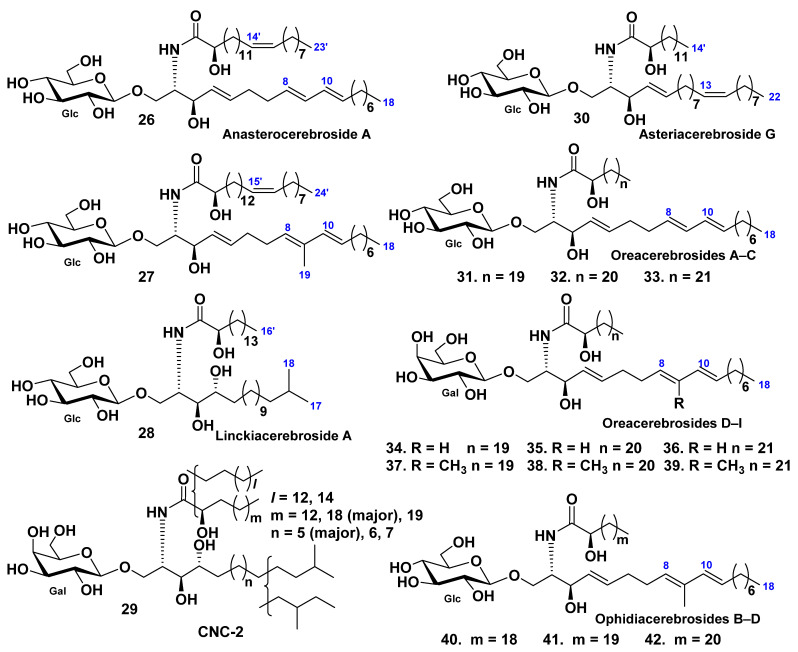
Structures of cerebrosides from the starfish *Anasterias minuta*, *Cosmasterias lurida*, *Linckia laevigata*, *Culcita novaeguineae*, *Oreaster reticulatus*, and *Narcissia canariensis*.

Eight glucosylceramides were found in the Patagonian starfish *Anasterias minuta* [43]. One of these constituents, anasterocerebroside A (**26**), was identified as a new glucosylceramide, while the earlier known glucosylceramide (**27**) was isolated and characterized for the first time as a pure compound. It was earlier isolated in a mixture with related glucosylceramides from the Patagonian starfish *Cosmasterias lurida* [40]. The structures of these sphingolipids were established by different spectroscopic and chemical methods as (2*S*,3*R*,4*E*,8*E*,10*E*)-1-*O*-(β-d-glucopyranosyl)-2-[(2*R*)-2-hydroxy-14-tricosenoylamino]-4,8,10-octadecatrien-3-ol (**26**) and (2*S*,3*R*,4*E*,8*E*,10*E*)-1-*O*-(β-d-glucopyranosyl)-2-[(2*R*)-2-hydroxy-15-tetracosenoylamino]-9-methyl-4,8,10-octadecatrien-3-ol (**27**) (Figure 4).

The glucocerebroside, linckiacerebroside A (**28**), and known glucocerebroside S-2a-3 were isolated from the chloroform–methanol extract of the starfish *Linckia laevigata*, together with three pseudo homogeneous glucocerebrosides [44]. The structures of this cerebroside were determined as (2*S*,3*S*,4*R*)-1-*O*-(β-d-glucopyranosyl)-2-[(2*R*)-2-hydroxyhexadecanoylamino]-16-methyl-heptadecane-1,3,4-triol (**28**) (Figure 4).

A galactocerebroside molecular species CNC-2 (**29**) were isolated from the extract of the tropical starfish *Culcita novaeguineae* [45] as a phytosphingosine type galactocerebroside with nonhydroxylated and hydroxylated fatty acyl moieties (Figure 4).

The glucocerebroside, asteriacerebroside G (**30**), and two known cerebrosides, asteriacerebrosides A and B, were isolated from the chloroform–methanol extract of the whole bodies of the Northern Pacific starfish *Asterias amurensis* [23]. The structure of **30** was determined on the basis of chemical and spectroscopic evidence as (2*S*,3*R*,4*E*,13*Z*)-1-*O*-(β-d-glucopyranosyl)-2-[(2*R*)-2-hydroxytetradecanoylamino]-4,13-docosadiene-1,3-diol (Figure 4). Asteriacerebrosides A, B, and G exhibited growth-promoting activity for the whole body of *Brassica campestris*.

The starfish *Oreaster reticulatus* contains nine glycosphingolipids named oreacerebrosides A–I (**31**–**39**) (along with earlier known ophidiacerebrosides C–E [46], Figure 4). All these compounds have a 4,8,10-triunsaturated sphingoid base. Oreacerebrosides A–C (**31**–**33**) are β-glucosylceramides in contrast with oreacerebrosides D–I (**34**–**39**), all these compounds were the first examples of β-galactosylceramides containing this unusual sphingoid base. Four representative glycosphingolipids were tested for cytotoxic activity on rat glioma C6 cells and shown to be mildly cytotoxic. Previously, it was established that the glucosylceramides were more active than the galactosylceramides. In addition, oreacerebroside I (**39**) was shown to exert proangiogenic activity and was able to increase VEGF-induced human endothelial cell proliferation.

Mixtures of three known glucocerebrosides (F13-3), ophidiacerebrosides B–D (**40**–**42**), were isolated from the starfish *Narcissia canariensis* collected off the coasts of Dakar, Senegal [47]. This fraction included three homologous cerebrosides identified as peracetylated derivatives on the basis of spectroscopic and chemical data (Figure 4). These compounds contain a β-glucopyranose as a sugar unit, 9-methyl-branched 4,8,10-triunsaturated long-chain aminoalcohols as sphingoid bases, and amide-linked 2-hydroxy fatty acid chains. The major component (63%) has an amide-linked 2-hydroxydocosanoic acid chain and was identified as ophidiacerebroside C (**41**), isolated from the starfish *Ophidiaster ophidianus* for the first time [48]. The minor components of F13-3 had one more or one less methylene group and were identified as ophidiacerebrosides B (**40**) and D (**42**). The cytotoxic activity of F13-3 was detected using KB cells. It was shown that three human cancerous cell lines, KMS-11 (adherent plasma cells obtained from patients with multiple myeloma) were inhibited by these cerebrosides with IC_50_ = 15.2 ± 4 μM, GBM (astrocytoma cells obtained after tumor resection of patients with glioblastoma multiforme-primary culture) with IC_50_ = 34.6 ± 5.1 μM, and HCT-116 (colorectal adenocarcinoma cells derived from a patient with Lynch’s syndrome) with IC_50_ = 18 ± 3.9 μM.

**Figure 5:**
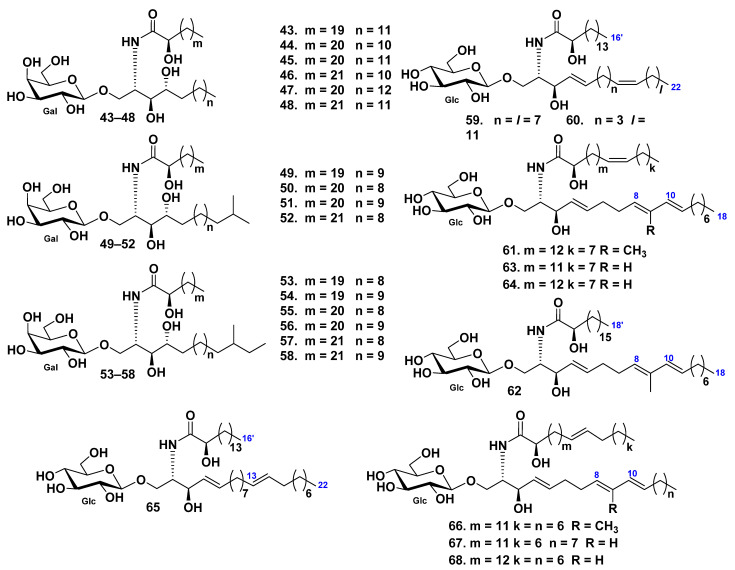
Cerebrosides from the starfish *Protoreaster nodosus*, *Asterias amurensis*, and *Distolasterias nipon*.

In total, 21 galactocerebrosides, including 16 new compounds (**43**–**58**) (Figure 5), were identified as cerebroside molecular species obtained from the chloroform–methanol extract of pyloric caeca cut out from the starfish *Protoreaster nodosus* [49]. These compounds were phytosphingosine-type galactocerebrosides with hydroxylated fatty acyl moieties. It is important, that GC–MS analysis, followed by methanolysis and periodate oxidation of these metabolites, gave reliable structural information of ceramide moiety rapidly in minute amounts. The structures of earlier known compounds were the same as those of galactosylcerebrosides previously found from other starfish and even mammalians.

Six glucocerebrosides (**59**–**64**) were isolated from the eggs of the starfish *Asterias amurensis* by extraction and different type of column chromatography, including HPLC [50]. It was shown that the structures of cerebrosides could be completely characterized, based on their sodium-adducted molecules, using FAB tandem mass spectrometry. The lipid part of the glucocerebrosides **59**–**64** consisted of saturated and monounsaturated α-hydroxy fatty acids and sphinganine type of the long-chain base (Figure 5).

Glucosyl ceramides (GlcCers) were later isolated from the viscera of the starfish *Asterias amurensis* [51]. Degraded GlcCers generated *A. amurensis* sphingoid bases (ASBs) that mainly consisted of the triene-type bases d18:3 and 9-methyl-d18:3. Actions of these bases on ceramide synthesis and content were analyzed using normal human epidermal keratinocytes (NHEKs). The bases significantly raised the de novo ceramide synthesis in NHEKs and expression of genes, encoding enzymes such as serinepalmitoyltransferase and ceramide synthase. Total ceramide (GlcCers) and sphingomyelin contents increased highly upon ASB treatment. In particular, GlcCers bearing fatty acids with large carbon atoms (≥ C28) exhibited a significant content increasing. These ASB-induced enhancements on de novo ceramide synthesis were only observed in undifferentiated NHEKs. This stimulation of de novo sphingolipid synthesis may improve skin barrier functions.

Four cerebrosides (**65**–**68**) were isolated from the starfish *Distolasterias nipon* by extraction and different type of column chromatography, including reverse-phase HPLC [34]. Structural elucidation was conducted using tandem mass spectrometry of monosodiated ions desorbed by fast atom bombardment. Fatty acids in glucocerebrosides **65**–**68** were identified as saturated and monounsaturated α-hydroxylated derivatives. The glucocerebroside long-chain bases were found to be of di- and triunsaturated sphingenine types (Figure 5).

### 3.2. Class Holothuroidea

**Figure 6:**
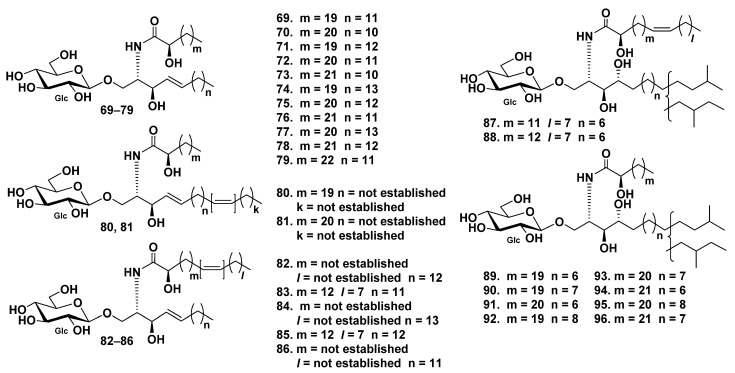
Cerebrosides from the sea cucumbers *Holothuria coronopertusa* and *Holothuria pervicax*.

Overall, 18 glucocerebrosides (**69**–**86**) were detected in admixture from the sea cucumber *Holothuria coronopertusa* [52]. Their structures were established on the basis of liquid-secondary ion mass spectrometry (LSIMS) experiments. The CID mass spectrum of the lithiated molecules ([M + Li]^+^) led to diagnostic fragment ions, which were further identified by tandem mass spectrometry (MS/MS). Fatty acids in glucocerebrosides **69**–**86** were indicated as saturated and monounsaturated α-hydroxyl fatty acids. The glucocerebroside long-chain bases were of sphingosine type (Figure 6).

Moreover, 10 glucocerebrosides, HPC-3-A–HPC-3-J (**87**–**96**), were isolated from the extract of the sea cucumber *Holothuria pervicax* [53]. All these compounds were mixtures of regio-isomers for terminal methyl groups in the LCB moiety, namely, mixtures of *iso*- and *anteiso*-isomers (Figure 6).

**Figure 7:**
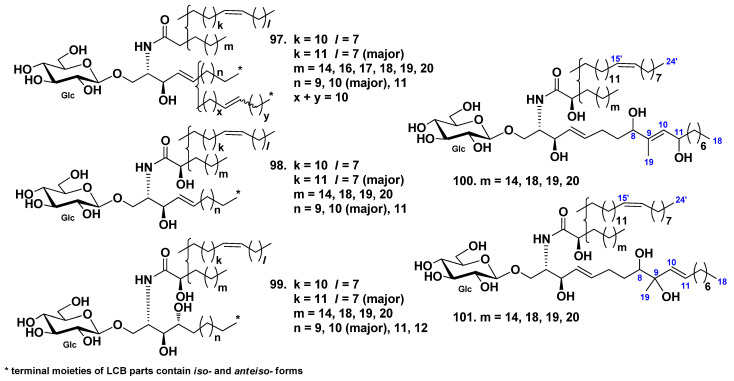
Cerebrosides molecular species from the sea cucumber *Stichopus japonicus*.

Five glucocerebroside molecular species (SJC-1-SJC-5, **97**–**101**) were isolated from the extract of the sea cucumber *Stichopus japonicus* [54]. Cerebrosides **97**–**99** were sphingosine- and phytosphingosine-type derivatives with nonhydroxylated and hydroxylated fatty acyl moieties. At the same time, cerebroside molecular species **100** and **101** were also sphingosine-type glucocerebroside molecular species with hydroxylated fatty acid moieties, although they were new compounds with unique sphingosine bases containing additional two hydroxy groups (Figure 7).

Later, the content and components of cerebrosides from the sea cucumber *Stichopus japonicus* were analyzed by Duan et al. [55]. The absorption of cerebrosides from *S. japonicus* was studied with an in vivo lipid absorption assay. The result revealed that *S. japonicus* was a rich source of cerebrosides that contained considerable amounts of odd carbon chain sphingoid bases. The cumulative recoveries of d17:1 and d19:2 consisting cerebrosides were 0.31 ± 0.16% and 0.32 ± 0.10%, respectively, for 24 h after administration. In addition, dietary supplementation with sea cucumber cerebrosides to hairless mouse improved the skin barrier function and increased the short-chain fatty acid content in caecal fraction, which demonstrated its effects on host.

**Figure 8:**
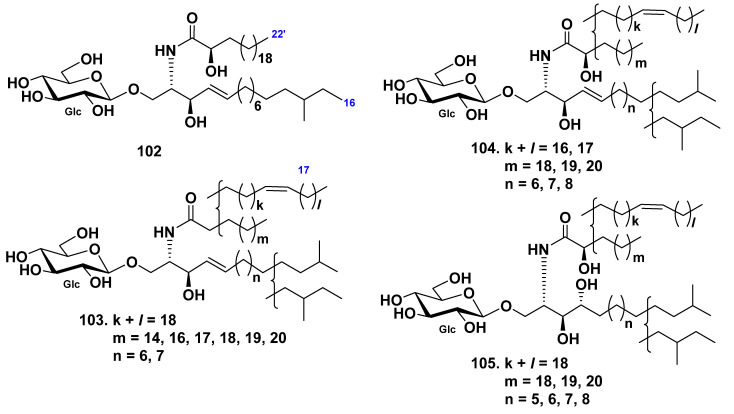
Cerebrosides from the sea cucumber *Holothuria leucospilota*.

An *anteiso*-type regio-isomer on the LCB moiety HLC-2-A (**102**) from the extract of the sea cucumber *Holothuria leucospilota* were isolated from its glucocerebroside molecular species HLC-2 (**104**), composed of *iso*- and *anteiso*-isomers [56]. Other glucocerebroside molecular species HLC-1(**103**) and HLC-3 (**105**) were indicated together with HLC-2 (Figure 8).

Sugavara et al. reported the sphingoid base composition of cerebrosides from sea cucumber (species was not identified) and their cytotoxicity against human colon cancer cell lines [57]. The composition of sphingoid bases obtained from a sea cucumber was different from that of mammals, and the major constituents were supposed from mass spectra as containing branched C-17–C-19 alkyl chains with 1–3 double bounds. The viability of DLD-1, WiDr, and Caco-2 cells treated with sea cucumber sphingoid bases was reduced in a dose-dependent manner and was similar to that of cells treated with sphingosine. The sphingoid bases induced such a morphological change as condensed chromatin fragments and increased caspase-3 activity, indicating that these sphingoid bases reduced the cell viability by causing apoptosis in the above-mentioned cells.

**Figure 9:**
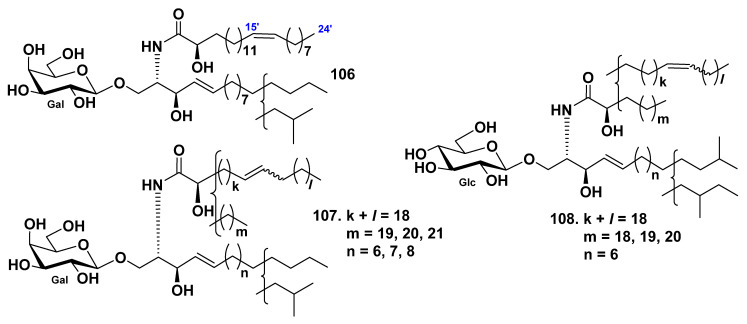
Cerebrosides from the sea cucumbers *Bohadschia argus* and *Acaudina molpadioides*.

The galactocerebroside BAC-4-4a (**106**) was isolated from its parent galactocerebroside molecular species BAC-4 (**107**), which was obtained from the extract of the sea cucumber *Bohadschia argus* [58]. BAC-4 was obtained together with earlier known glucocerebroside molecular species [53,54,56]. The structure of **106** was determined as (2*S*,3*R*,4*E*)-1-*O*-(β-d-galactopyranosyl)-2[(2*R*,15*Z*)-2-hydroxytetracosenoylamino]-4-heptadecene-1,3-diol (Figure 9). Before this study, galactocerebrosides were not found in sea cucumbers.

The cerebroside molecular species AMC-2 (**108**) was isolated from the extract of the sea cucumber *Acaudina molpadioides* [59]. The amide-linked fatty acid units were established to contain four saturated and monounsaturated α-hydroxy fatty acids, the long-chain dihydroxy sphingoid base, having one double bond, and the glucose residue (Figure 9). It was shown the anti-fatty liver activity of 108 in rats with fatty liver, induced by orotic acid. AMC-2 (**108**) significantly reduced hepatic triglyceride and total cholesterol levels at a diet supplement of 0.03% and 0.006%. The indexes of stearoyl–CoA desaturase activity and mRNA expression were significantly decreased by **108**. This indicated that AMC-2 (**108**) ameliorated nonalcoholic fatty liver disease through suppression of stearoyl–CoA desaturase activity and impaired the biosynthesis of monounsaturated fatty acids in the livers of the rats.

Glucocerebrosides from three specimens of sea cucumbers, specifically, *Acaudina molpadioides*, *Cucumaria frondosa*, and *Apostichopus japonicus*, were rapidly identified by liquid chromatography–ion trap–time-of-flight mass spectrometry [60]. Various long-chain bases of glucosylcerebrosides were detected in these sea cucumbers. Two of the most common LCBs were identified as 2-amino-1,3-dihydroxy-4-heptadecene (d17:1) and 4,8-sphingadienine (d18:2), which were acylated to form saturated and monounsaturated nonhydroxylated and monohydroxylated fatty acids with 18–25 carbon atoms. The glucocerebroside fractions were the most complicated in the sea cucumber *C. frondosa* and were the simplest in the sea cucumber *A. molpadioides*.

It was found that a continuous oral administration of cerebrosides obtained from the sea cucumber *Acaudina molpadioides* at the dose of 50 mg/kg body mass per day suppressed body weight loss through alleviating adipose atrophy in cancer-associated cachexia mice [61]. The long-chain base, hydrolyzed from the cerebroside, contains 2-amino-1,3-dihydroxy-4-heptadecene (d17:1), which is a typical predominant sphingoid base in sea cucumbers. The possible mechanism by which dietary cerebrosides prevent adipose atrophy in cancer-associated cachexia mice was related to reducing serum inflammatory cytokine levels, regulating over lipolysis, enhancing the function of lipogenesis, and decreasing the lipid over-utilization. To elucidate the structure–activity relationships of cerebrosides and their long-chain base, the antitumor activities were compared between them. The results indicated that LCBs exhibited a more prominent antitumor effect both in vivo and in vitro.

In addition, sea cucumber cerebrosides and their main structural units, long-chain bases, were obtained from *Acaudina molpadioides* and then administered to high fat diet-induced obese C57BL/6J mice at a diet supplement dosage of 0.025% for 5 weeks to evaluate their effects on obesity-related metabolic disorders [62]. Cerebrosides and long-chain bases significantly decreased epididymal adipose tissue weights, lowered hepatic triacylglycerol levels, and reduced serum glucose, insulin levels, and insulin resistance HOMA-IR index in mice. The activities of hepatic lipogenetic proteins including FAS, ME, and the mRNA levels encoding proteins SREBP-1c and FAS were reduced by cerebrosides and long-chain bases treatment. However, cerebrosides and LCBs showed no effect on the hepatic lipolysis pathway. Moreover, cerebrosides and LCBs efficiently upregulated the gene expression of SREBP-1c, FAS, ACC, ATGL, and HSL, and downregulated the gene expression of LPL and VLDL-r in the adipose tissue. These results demonstrated that cerebrosides and LCBs were effective in suppressing hepatic SREBP-1c mediated lipogenesis, inhibiting lipid uptake, and increasing TG catabolism in the adipose tissue. The ameliorative degree and regulatory mechanisms of these two groups of natural products were basically the same, suggesting that long-chain bases are the key active structural units of cerebrosides [62].

**Figure 10:**
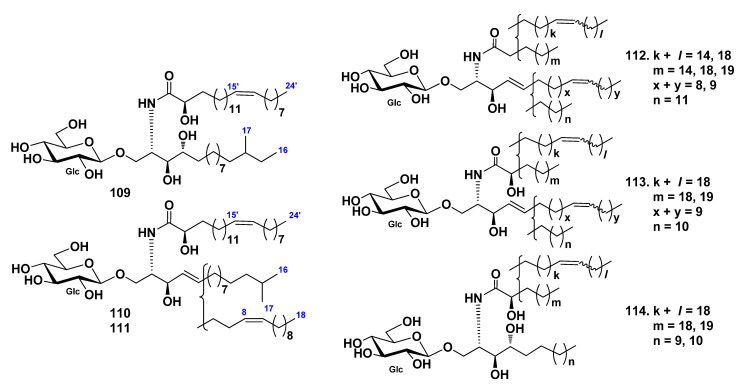
Cerebrosides from the sea cucumber *Cucumaria frondosa*.

Three glucocerebrosides, CF-3-1, CF-3-2, and CF-3-3 (**109**–**111**), were isolated from the cerebroside fraction, which was obtained from the chloroform–methanol extract of the sea cucumber *Cucumaria frondosa* by La et al. [63]. The structures of these cerebrosides were determined as 1-*O*-β-d-glucopyranosides of (2*S*,3*S*,4*R*)-2-[(2*R*,15*Z*)-2-hydroxy-15-tetracosenoylamino]-14-methylhexadecane-1,3,4-triol (**109**), (2*S*,3*R*,4*E*)-2-[(2*R*,15*Z*)-2-hydroxy-15-tetracosenoylamino]-15-methyl-4-hexadecene-1,3-diol (**110**), and (**111**) (2*S*,3*R*,4*E*,8*Z*)-2-[(2*R*,15*Z*)-2-hydroxy-15-tetracosenoylamino]-4,8-octadecadiene-1,3-diol (Figure 10). Compounds **110** and **111** were obtained as pure compounds for the first time.

Three glucocerebroside molecular species (CFC-1, CFC-2, and CFC-3, **112**–**114**) were isolated from total cerebrosides from the sea cucumber *Cucumaria frondosa* by Xu et al. (Figure 10) [64]. The structures of these substances were elucidated on the basis of spectroscopic and chemical evidence: fatty acids were identified mainly as saturated (C22:0 and C18:0), monounsaturated (C24:1 and C20:1), and α-hydroxylated derivatives (C24:1h, C23:0h, C23:1h, and C22:0h), the LCB were identified as dihydroxy (d17:1, d18:2, and d18:1) and trihydroxy (t17:0 and t16:0) compounds. The composition analysis of long-chain bases showed that the ratio of d18:2 and d17:1 was approximately 2:1. Four glucocerebrosides and long-chain bases from sea cucumber *Cucumaria frondosa* were evaluated for their cytotoxic activities against Caco-2 colon cancer cells in in vitro assays. The obtained results indicated that both glucocerebrosides and LCB demonstrated an inhibitory effect on cell proliferation. Moreover, **114** was the most effective substance from these four glucocerebrosides in the Caco-2 cell viability test. The inhibitory effects of long-chain bases were much stronger than glucocerebrosides.

Glucocerebrosides, isolated from the sea cucumber *Cucumaria frondosa* (CFC), were investigated on their antiadipogenic activity in vitro [65]. These glucocerebrosides inhibited the lipid accumulation of 3T3-L1 cells and suppressed PPARγ and C/EBPα expressions, which confirmed their antiadipogenic effect. Furthermore, CFCs suppressed lipogenesis in mature adipocytes. Glucocerebrosides enhanced β-catenin expression, promoted its nuclear translocation, and upregulated the expression of CCND1 and c-myc, two target genes of β-catenin. Moreover, after cells were treated with the β-catenin inhibitor 21H7, β-catenin nuclear translocation and transcription activity can be recovered by CFC. These findings suggested that glucocerebrosides from *Cucumaria frondosa* promoted the activation of the WNT/β-catenin pathway. Additionally, CFCs enhanced the expressions of Wnt-receptor frizzled-like protein variant 1(FZ1), low-density lipoprotein receptor-related proteins LRP5, and LRP6, while they had no effect on the expressions of Wnt10b and GSK3β proteins. These findings also confirmed that glucocerebrosides exhibit their antiadipogenic activity through enhancing the activation of the WNT/β-catenin pathway, which was mediated by FZs and LRPs.

Over the past two decades, about a hundred individual cerebrosides and their molecular species were isolated from starfish and sea cucumbers. The isolated compounds contain both sphingosine and phytosphingosine bases of *normal*-, *iso*- and *anteiso*-types. In most cases, long-chain bases include from 16 to 19 carbon atoms, but there were also longer ones, up to C-22. In addition, many LCBs were unsaturated and contained one or two double bonds. In particular, (4*E*,8*E*,10*E*)-sphinga-4,8,10-trienine; (4*E*,8*E*,10*E*)-9-methyl-sphinga-4,8,10-trienine; (4*E*,13*Z*)-sphinga-4,13-dienine; (4*E*,15*Z*)-sphinga-4,15-dienine; and (9*Z*)-4-hydroxy-9-sphingenine long-chain bases were often found. At the same time, unique oxidized LCB (4*E*,9*E*)-9-methyl-8,11-dihydroxy-sphinga-4,9-dienine, and (4*E*,10*E*)-9-methyl-8,9-dihydroxy-sphinga-4,10-dienine were found in the sea cucumber *Stichopus japonicus*.

In most cases, the fatty acids in the cerebrosides were long-chain C-22–C-24 (2*R*)-2-hydroxy acids of *normal*-, *iso*-, and *anteiso*-types. However, shorter FAs such as C-18, C-16, and even C-14 were also found. Some fatty acids in the isolated cerebrosides were unsaturated and most of them had the (15*Z*)-double bond. In contrast to cerebrosides from starfish, cerebrosides from sea cucumbers contained non-α-hydroxylated FA with different long polymethylene chains.

The carbohydrates in cerebrosides of starfish and sea cucumbers were represented by the β-d-glucopyranose and, more rarely, the β-d-galactopyranose. Thus far, no other types of monosaccharide residues have been found in cerebrosides of starfish and sea cucumbers. In addition, cerebrosides lactosides (with Gal-(1→4)-Glc-(1→1)-Cer moieties) were isolated from the starfish *Luidia maculata*. Other variants of cerebroside biglycosides or oligoglycosides in starfish and sea cucumbers have not been found.

The following types of biological activity of cerebrosides from starfish and sea cucumbers were established: *i.* growth-promoting activity on *Brassica campestris*, *ii.* cytotoxic activity against epidermal carcinoma of the mouth KB cells and rat glioma C6 cells; and *iii.*proangiogenic activity. More detailed data are given in Table 1. The conducted studies showed the promising prospects of the practical use of cerebrosides of starfish and sea cucumbers. Accordantly, further expansion of the studies on the biological activity of this class of glycolipids is required, as well as additional data concerning the molecular mechanisms of their action.

## 4. Gangliosides

Gangliosides are known as additionally hydroxylated derivatives of cerebrosides with one or more sialic acid residues in their carbohydrate chains. Sialic acids are a group of higher carbohydrates with nine carbon atoms, which includes several dozens of derivatives of neuraminic acid (NeuAc) [87]. Gangliosides were so named for the first time because they were isolated from brain ganglion cells. It is considered that gangliosides are metabolites of vertebrates; however, they were also found in all classes of Echinoderms and may indicate a high organization of their nervous system. To designate gangliosides, they most often use abbreviated names according to Svennerholm’s nomenclature, in which gangliosides are divided into so-called series, indicated by the number of sialic acid units and their position in the carbohydrate chain. Gangliosides are biosynthesized from the corresponding cerebrosides by sialyltransferases on the inner plasma membrane or in the Golgi apparatus, and then they are incorporated into the plasmatic membrane, where these glycosphingolipids perform their biological functions [88]. Gangliosides play an important role in binding to some lectins and affect the activity of receptor protein kinases, taking part in the transmission of cellular signals. In addition, gangliosides, similar to other sphingolipids and cholesterol, play an important role in stabilizing plasma membranes with positive curvature and also affect the surface charge of the membrane. Finally, gangliosides can act as receptors for viruses, bacteria, and toxins, thus being part of the immune system [88].

It is known that gangliosides play an extremely important role in the development of various neurodegenerative diseases, as well as in the regulation of proliferation and energy metabolism of tumor cells [89,90,91].

Thus, the search for new structural types of gangliosides in echinoderms, as well as a comprehensive study of their biological activity, is an actual scientific task.

### 4.1. Class Asteroidea

**Figure 11:**
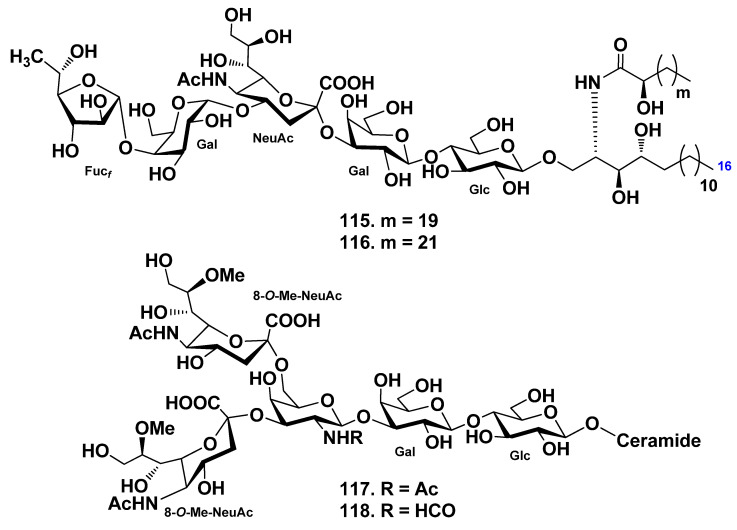
Gangliosides from the starfish *Acanthaster planci* and *Evasterias echinosoma*.

The ganglioside molecular species, AG-1, were obtained from the whole body of the starfish *Acanthaster planci* [70]. Enzymatic hydrolysis by endoglycoceramidase gave an oligosaccharide and ceramides, quantitatively. The oligosaccharide moiety was determined mainly by 2D-NMR experiments as β-Fuc_f_-(1→4)-α-Gal_p_-(1→4)-α-NeuAc-(2→3)-β-Gal_p_-(1→4)-Glc_p_. The sphingoid moiety was elucidated as the mixture of (2*S*,2’*S*,3*S*,4*R*)-2-((2*R*)-2-hydroxydocosanoylamino)-1,3,4-trihydroxyhexadecane and (2*S*,2’*S*,3*S*,4*R*)-2-((2*R*)-2-hydroxytetracosanoylamino)-1,3,4-trihydroxyhexadecane. Reversed-phase HPLC of AG-1 gave two kinds of gangliosides named acanthagangliosides I (**115**) and J (**116**). It is clear that the oligosaccharide moiety of AG-1 is different in its terminal monosaccharide when compared with AG-2 and AG-3, which were isolated from *A. planci* earlier [71,72]. The terminal β-Gal_f_ of AG-2 and AG-3 is linked to C-3 of α-Gal_p_, while the terminal β-Fuc_f_ of AG-1 is linked to C-4 of α-Gal_p_. This interesting difference in terminal sugar linkages seems to be derived from the coexistence of different glycosyltransferases, namely, β-1,3-galactofuranosyl transferase and β-1,4-fucofuranosyl transferase. The gangliosides of *A. planci* characteristically have a terminal furanose-type sugar unit (Figure 11).

It was found by performing ^1^H NMR and saturation transfer difference (STD) NMR experiments that AG2 pentasaccharide (structure not shown) binds to human Siglec-2 (a mammalian sialic acid-binding protein expressed on B-cell surfaces, which involved in the modulation of B-cell mediated immune response [73]. STD NMR experiments indicated that the C-7–C-9 carbohydrate-chain and the acetamide moiety of the central sialic acid residue were located in the binding face of human Siglec-2. The binding epitope of AG2 pentasaccharide to human Siglec-2 was determined as the α-Gal_p_(1→4)-α-NeuAc-(2→3)-Gal_p_ unit. The information concerning the binding epitope of AG2 pentasaccharide is of value toward the development of potent Siglec-2 inhibitors.

Gangliosides molecular species were isolated from the starfish *Evasterias echinosoma*, and their structures were elucidated [66]. Two major sphingolipids (**117**, **118**) were found to be disialogangliosides, whose carbohydrate chain is based on the trisaccharide β-*N*-acylgalactopyranosaminyl-(1→3)-β-galactopyranosyl-(1→4)-β-glucopyranose (acyl is formyl or acetyl). Both residues of 8-*O*-methyl-*N*-acetylneuraminic acid are attached to the *N*-acylgalactosamine residue at positions C-3 and C-6. Compound **118** is the first example of when an *N*-formyl derivative of an amino sugar was found in gangliosides. The lipid part of the gangliosides molecular species consists of monounsaturated sphingoid base and nonhydroxylated fatty acids (mainly, palmitic and stearic acids) (Figure 11).

**Figure 12:**
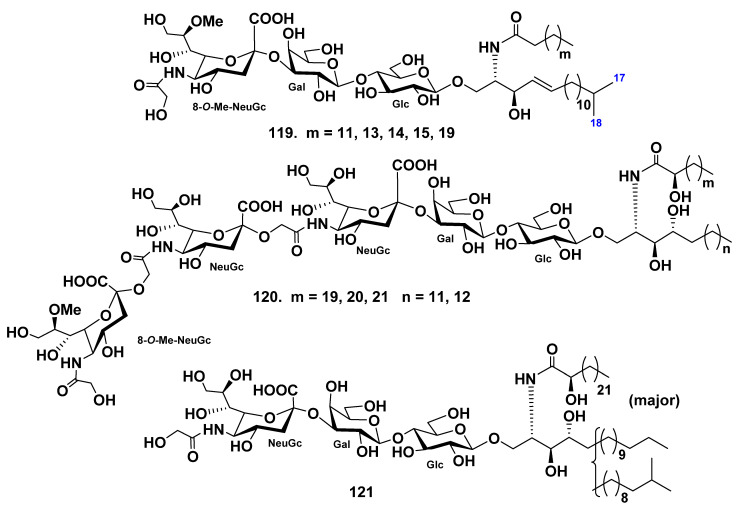
Gangliosides from the starfish *Linckia laevigata*.

The ganglioside (**119**) was isolated from the starfish *Linckia laevigata*, and its structure was determined by spectroscopic and chemical methods [75]. The carbohydrate part was proved to be 8-*O*-Me-(*N*-glycolyl-α-d-neuraminosyl)-(2→3)-β-d-galactopyranosyl-(1→4)-β-d-glucopyranoside. The lipid moiety of this ganglioside consists of nonhydroxylated fatty acids (the major component is palmitic acid) and *iso*-C18:1-sphingenine. Based on the structure of the carbohydrate moiety, ganglioside **119** belongs to the hematoside type, characteristic of erythrocytes of vertebrates. It differs from the other known hematosides in the nature of the sialic acid. A hematoside with 8-*O*-methyl-*N*-glycolylneuraminic acid unit was found for the first time (Figure 12).

Continuing research on gangliosides of the starfish *Linckia laevigata,* ganglioside molecular species LLG-5 (**120**) were obtained from the water-soluble portion of its lipid fraction [76]. On the basis of spectroscopic and chemical data, the structure of **120** was elucidated as 8-*O*-methyl-(*N*-glycolyl-α-d-neuraminosyl)-(2→11)-(*N*-glycolyl-α-d-neuraminosyl)-(2→11)-(*N*-glycolyl-α-d-neuraminosyl)-(2→3)-β-d-galactopyranosyl-(1→4)-β-d-glucopyranoside of a ceramide composed of phytosphingosines and 2-hydroxy *n*-fatty acids. The major components of the fatty acids and long-chain bases moieties of 120 were identified as (2*R*)-2-hydroxy *n*-docosanoic acid and (2*S*,3*S*,4*R*)-2-amino-1,3,4-octadecanetriol, respectively. This was the first isolation and characterization of a trisialo-ganglioside from Asteroidea (Figure 12). Furthermore, **120** is a new ganglioside molecular species containing a 2→11 linked trisialosyl moiety. The ganglioside molecular species LLG-5 (**120**) exhibited neuritogenic activity in rat pheochromocytoma PC12 cells in the presence of nerve growth factor (NGF). The proportion of cells with neurites longer than the diameter of the cell body at a concentration of 10 μM or 120 was 59.3% when compared with the control (NGF, 5 ng/mL: 20.6%). Furthermore, their effect was greater than that of the mammalian ganglioside GM1 (47.0%).

In addition, the hematoside-type ganglioside LLG-1 (**121**) was obtained from the polar lipid fraction of the starfish *Linckia laevigata* [77]. The structure of LLG was elucidated on the basis of spectroscopic and chemical evidence as 1-*O*-[(*N*-glycolyl-α-d-neuraminosyl)-(2→3)-β-d-galactopyranosyl-(1→4)-β-d-glucopyranosyl]-ceramide. The ceramide moiety was composed of 2-hydroxy fatty acids and phytosphingosine units (*normal*- and *iso*-type long-chain bases). This was the first report on the isolation and structure elucidation of naked hematoside-type ganglioside from echinoderms (Figure 12).

**Figure 13:**
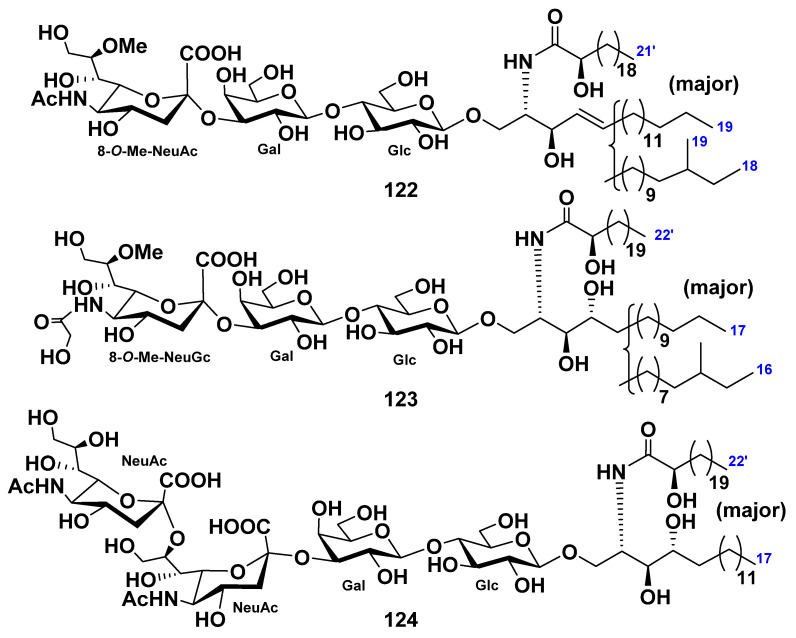
Ganglioside molecular species from the starfish *Luidia maculata*.

Two monomethylated GM3-type ganglioside molecular species (**122** and **123**) were isolated from the extract of the starfish *Luidia maculata* [68]. The structures of these gangliosides were determined as 1-*O*-[8-*O*-methyl-(*N*-acetyl-α-d-neuraminosyl)-(2→3)-β-d-galactopyranosyl-(1→4)-β-d-glucopyranosyl]-ceramide (**122**) and 1-*O*-[8-*O*-methyl-(*N*-glycolyl-α-d-neuraminosyl)-(2→3)-β-d-galactopyranosyl-(1→4)-β-d-glucopyranosyl]-ceramide (**123**). The ceramide moieties were composed of heterogeneous nonhydroxylated fatty acid, 2-hydroxy fatty acid, sphingosine, and phytosphingosine units. Compound **122**, designated as LMG-3, represented new ganglioside molecular species. Compound **123** was identified as a known ganglioside molecular species (Figure 13).

In addition, the GD3-type ganglioside molecular species LMG-4 (124) was obtained from the extract of the starfish *L. maculata* [69]. The structure of this compound was determined on the basis of spectroscopic and chemical evidence to be 1-*O*-[(*N*-acetyl-α-d-neuraminosyl)-(2→8)-(*N*-acetyl-α-d-neuraminosyl)-(2→3)-β-d-galactopyranosyl-(1→4)-β-d-glucopyranosyl]-ceramide. The ceramide moiety was composed of 2-hydroxy fatty acid and phytosphingosine moieties. GD3-type ganglioside was isolated and its particular structure elucidated for the first time from echinoderms (Figure 13). LMG-4 (**124**) exhibited neuritogenic activity toward the rat pheochromocytoma PC12 cells in the presence of NGF. The proportion of the neurite-bearing cells of **124** at a concentration of 10 μM was 47.7%, in comparison with the control (NGF, 5 ng/mL: 20.6%). The effect of **124** was the same as that of the mammalian ganglioside GM1 (47.0%).

**Figure 14:**
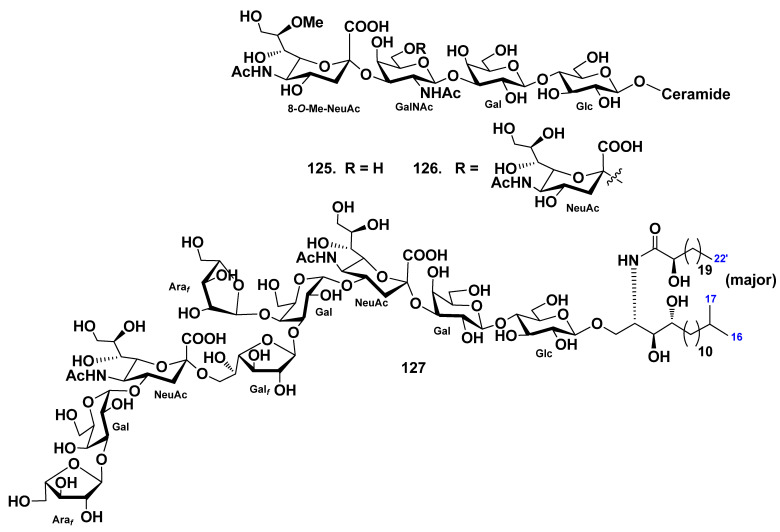
Gangliosides from the starfish *Evasterias retifera* and *Patiria (*=*Asterina) pectinifera*.

Mono- and disialogangliosides (**125**, **126**) were isolated from gonads of the starfish *Evasterias retifera* [67]. Their structures were elucidated by spectroscopic and chemical evidence, including enzymatic hydrolysis with neuraminidase. The monosialoganglioside has the structure α-8-*O*-Me-NeuGc-(2→3)-β-GalNAc-(1→3)-β-Gal-(1→4)-β-Glc-(1→1)-Cer, while the disialoganglioside contains an additional NeuAc residue, which glycosylates GalNAc in position C-6. The lipid moieties of both gangliosides contain phytosphingosine bases (mainly C18:0) and two types of fatty acids, nonhydroxylated (mainly C16:0 and C18:0) and α-hydroxylated (mainly α-hydroxy-C16:0) (Figure 14).

The molecular species GP-3 (**127**) was obtained from the starfish *Patiria (=Asterina) pectinifera* [74]. The structure of the ganglioside was determined as 1-*O*-α-l-arabinofuranosyl-(1→3)-α-d-galactopyranosyl-(1→4)-(*N*-acetyl-α-d-neuraminosyl)-(2→6)-β-d-galactofuranosyl-(1→3)-[α-l-arabinofuranosyl-(1→4)]-α-d-galactopyranosyl-(1→4)-(*N*-acetyl-α-d-neuraminosyl)-(2→3)-β-d-galactopyranosyl-(1→4)-β-d-glucopyranoside of ceramide composed of heterogeneous (2*S*,3*S*,4*R*)-phytosphingosine (*iso*-C-17-phytosphingosine as the major component) and (2*R*)-2-hydroxy fatty acid units (docosanoic acid as the major component) (Figure 14). Compound **127** represents new ganglioside molecular species possessing two residues of sialic acids at the inner part of the sugar moiety. A ganglioside molecular species GP-3 (**127**) exhibits neuritogenic activity toward the rat pheochromocytoma cell line PC12, in the presence of NGF. The proportion of the cells with neurite longer than the diameter of the cell body at the use of **127** at a concentration of 10 μM was 38.2% when compared with the control (NGF, 5 ng/mL: 20.6%). The effect of **127** was lower than that of the mammalian ganglioside GM1 (47.0%).

**Figure 15:**
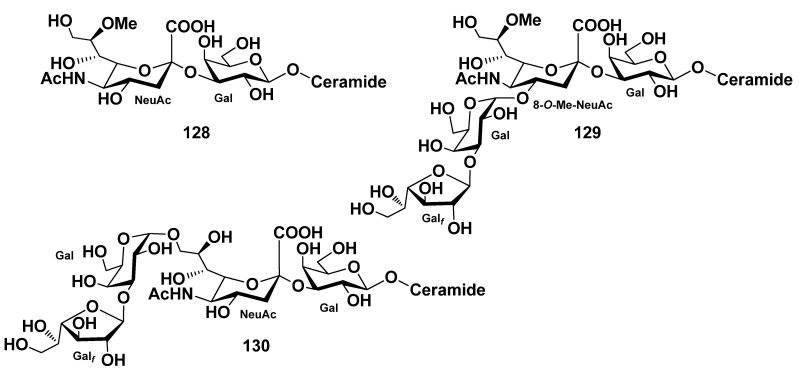
Gangliosides molecular species from the starfish *Protoreaster nodosus*.

Three ganglioside molecular species PNG-1 (**128**), PNG-2A (**129**), and PNG-2B (**130**) were isolated from pyloric caeca of the starfish *Protoreaster nodosus* [78]. Their structures as 1-*O*-[8-*O*-methyl-(*N*-acetyl-α-neuraminosyl)-(2→3)-β-galactopyranosyl]-ceramide (**128**), 1-*O*-[β-galactofuranosyl-(1→3)-α-galactopyranosyl-(1→4)-8-*O*-methyl-(*N*-acetyl-α-neuraminosyl)-(2→3)-β-galactopyranosyl]-ceramide (**129**), and 1-*O*-[β-galactofuranosyl-(1→3)-α-galactopyranosyl-(1→9)-(*N*-acetyl-α-neuraminosyl)-(2→3)-β-galactopyranosyl]-ceramide (**130**) were elucidated by a combination of spectroscopic and chemical methods. The ceramide moieties of ganglioside molecular species consisted of (2*S*,3*S*,4*R*)-phytosphingosines (*iso*-C-18-phytosphingosine as the major component) and (2*R*)-2-hydroxy fatty acid units (docosanoic acid as the major component). PNG-2A (**129**) and PNG-2B (**130**) represent the first GM4 elongation products in nature (Figure 15).

### 4.2. Class Holothuroidea

**Figure 16:**
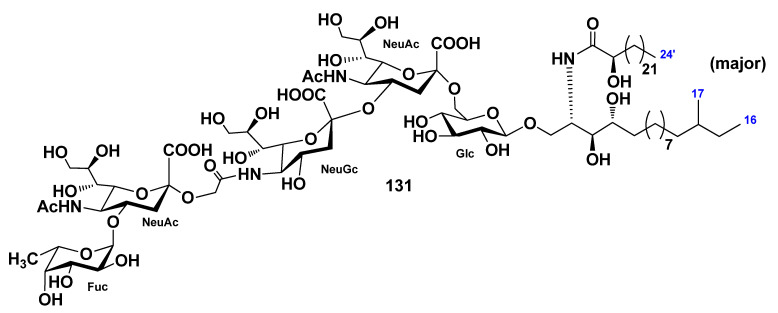
Ganglioside molecular species from the sea cucumber *Holothuria pervicax*.

The ganglioside molecular species HPG-7 (**131**) was isolated from the chloroform–methanol extract of the sea cucumber *Holothuria pervicax* [79]. On the basis of the spectroscopic and chemical evidence, the structure of the major component of **131** was determined as 1-*O*-[α-l-fucopyranosyl-(1→4)-(*N*-acetyl-α-d-neuraminosyl)-(2→11)-(*N*-glycolyl-α-d-neuraminosyl)-(2→4)-(*N*-acetyl-α-d-neuraminosyl)-(2→6)-β-d-glucopyranosyl]-(2*S*,3*S*,4*R*)-[(2*R*)-2-hydroxytetracosanoylamino]-14-methyl-hexadecane-1,3,4-triol (Figure 16). The trisialo-ganglioside was isolated for the first time from sea cucumbers. HPG-7 (**131**) was studied for neuritogenic action toward the PC12 rat pheochromocytoma cell line. It was shown that **131** does not have neuritogenic activity, in comparison with control, at a concentration of above 10 μg/mL, similar to three other ganglioside molecular species (HPG-1, HPG-3, and HPG-8) [80].

**Figure 17:**
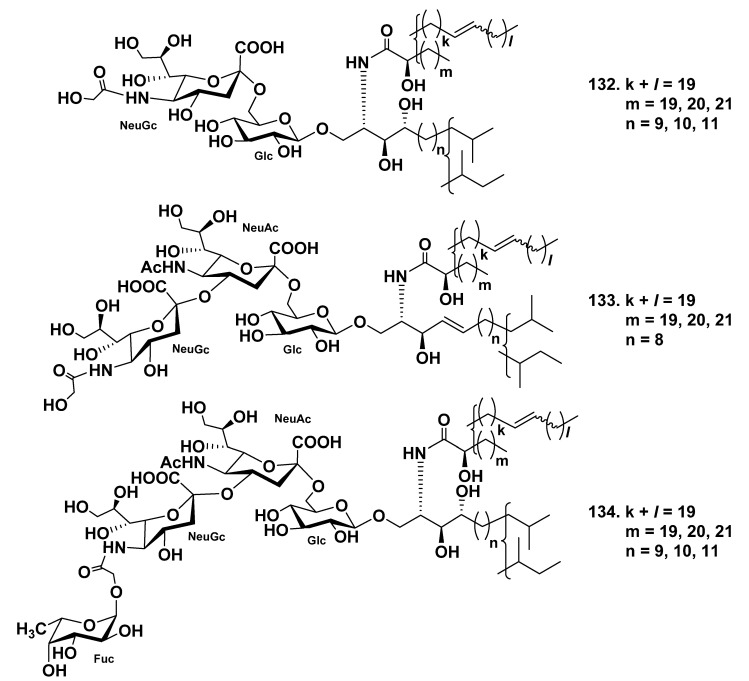
Ganglioside molecular species from the sea cucumber *Holothuria* *leucospilota*.

Three ganglioside molecular species, HLG-1 (**132**), HLG-2 (**133**), and HLG-3 (**134**), were isolated from the extract of the sea cucumber *Holothuria leucospilota* [81]. Structures of these gangliosides were determined as 1-*O*-[(*N*-glycolyl-α-d-neuraminosyl)-(2→6)-β-d-glucopyranosyl]-ceramide (**132**), 1-*O*-[(*N*-glycolyl-α-d-neuraminosyl)-(2→4)-(*N*-acetyl-α-d-neuraminosyl)-(2→6)-β-d-glucopyranosyl]-ceramide (**133**), and 1-*O*-[α-l-fucopyranosyl-(1→11)-(*N*-glycolyl-α-d-neuraminosyl)-(2→4)-(*N*-acetyl-α-d-neuraminosyl)-(2→6)-β-d-glucopyranosyl]-ceramide (**134**), respectively. The ceramide moieties were composed of phytosphingosines or sphingosines and 2-hydroxy fatty acids (Figure 17). Compounds **133** and **134** represent new ganglioside molecular species. These three substances showed slight neuritogenic activity toward the rat pheochromocytoma cell line PC12 cell in the presence of NGF.

**Figure 18:**
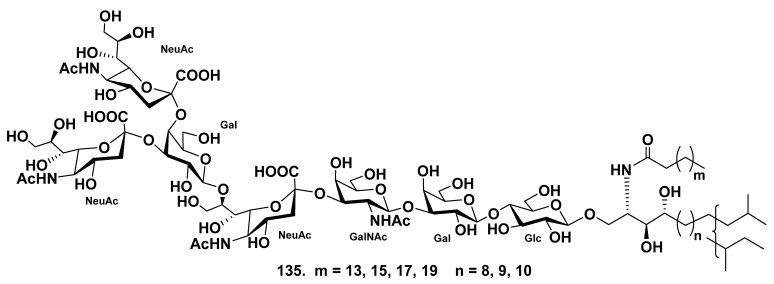
Ganglioside molecular species from the sea cucumber *Stichopus japonicus*.

The ganglioside molecular species SJG-2 (**135**) was obtained from the extract of the sea cucumber *Stichopus japonicus* [82]. On the basis of spectroscopic and chemical studies, the structure of SJG-2 (**135**) was determined as α-NeuAc-(2→4)-α-NeuAc-(2→3)-β-Gal-(1→8)-α-NeuAc-(2→3)-β-GalNAc-(1→3)-β-Gal-(1→4)-β-Glc-(1→1)-Cer. The ganglioside **135**, possessing a unique carbohydrate moiety, is the first corresponding substance with a branched sugar chain moiety and *N*-acetylgalactosamine residue isolated from sea cucumbers (Figure 18). Ganglioside SJG-2 (**135**) exhibited neuritogenic activity toward the rat pheochromocytoma cell line PC12 cells in the presence of NGF. The proportion of neurite-bearing cells at the use of SJG-2 (64.8 ± 7.6%) was larger than that induced by the previously isolated SJG-1 [83], (35.4 ± 4.0%) when compared with the control (NGF, 5 ng/mL: 20.6 ± 2.2%). Furthermore, the effect of SJG-2 (**135**) was more considerable than that of the mammalian ganglioside GM1 (47.0 ± 2.5%).

**Figure 19:**
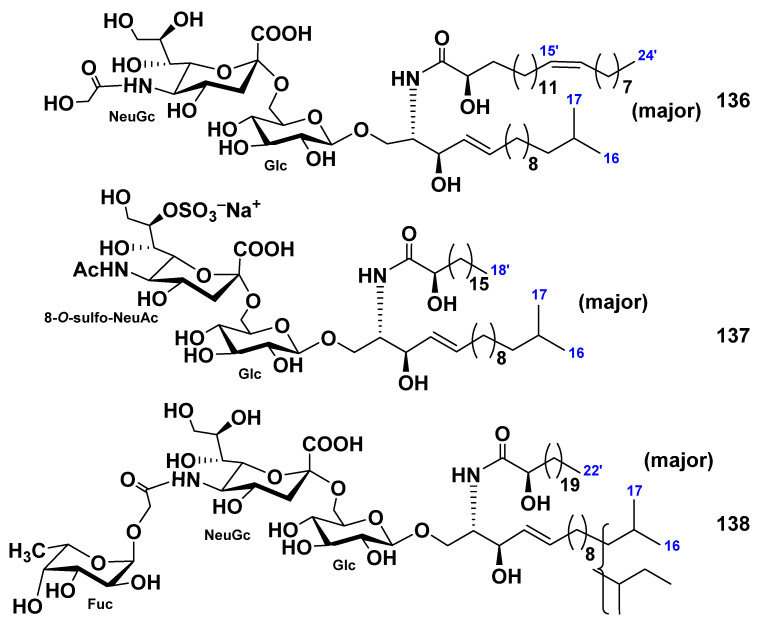
Ganglioside molecular species from the sea cucumber *Stichopus chloronotus*.

Three ganglioside molecular species, SCG-1 (**136**), SCG-2 (**137**), and SCG-3 (**138**), were isolated from the extract of the sea cucumber *Stichopus chloronotus* [84]. On the basis of spectroscopic and chemical evidence, the structures of these gangliosides were determined to be 1-*O*-[(*N*-glycolyl-α-d-neuraminosyl)-(2→6)-β-d-glucopyranosyl]-ceramide (**136**), 1-*O*-[8-*O*-sulfo-(*N*-acetyl-α-d-neuraminosyl)-(2→6)-β-d-glucopyranosyl]-ceramide (**137**), and 1-*O*-[α-l-fucopyranosyl-(1→11)-(*N*-glycolyl-α-d-neuraminosyl)-(2→6)-β-d-glucopyranosyl]-ceramide (**138**). The ceramide moieties were composed of isomeric long-chain bases and fatty acid units. The molecular species **138** is the first representative of gangliosides containing fucopyranose in the sialosyl trisaccharide moiety (Figure 19). Gangliosides **136**–**138** exhibited neuritogenic activity toward the rat pheochromocytoma PC12 cells in the presence of NGF. The proportions of the neurite-bearing cells at a concentration of **136**–**138** of 3.3 μg/mL were 34.1%, 24.4%, and 24.5%, respectively. These effects were compared with that of the mammalian ganglioside GM1 (22.1% at a concentration of 3.3 mg/mL).

**Figure 20:**
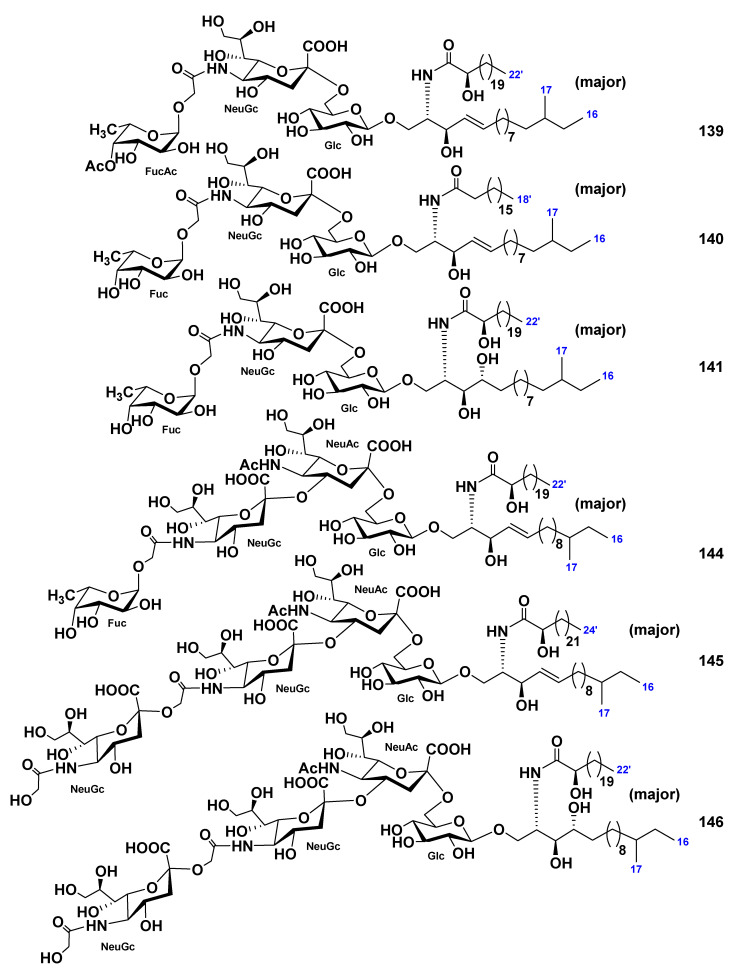
Gangliosides from the sea cucumber *Cucumaria echinata*.

Three monosialo-gangliosides, CEG-3 (**139**), CEG-4 (**140**), and CEG-5 (**141**), were obtained, together with two previously known gangliosides, SJG-1 (**142**, structure not shown, [83]) and CG-1 (**143**, structure not shown, [92]), from the extract of the sea cucumber *Cucumaria echinata* [85]. In addition, three disialo- or trisialo-gangliosides, CEG-6 (**144**), CEG-8 (**145**), and CEG-9 (**146**)**,** were also obtained along with the known ganglioside, HLG-3 (**134**, [81]) from this species of sea cucumbers [86]. Structures of these gangliosides were determined as 1-*O*-[(4-*O*-acetyl-α-l-fucopyranosyl)-(1→11)-(*N*-glycolyl-α-d-neuraminosyl)-(2→6)-β-d-glucopyranosyl]-ceramide (**139**), 1-*O*-[α-l-fucopyranosyl-(1→11)-(*N*-glycolyl-α-d-neuraminosyl)-(2→6)-β-d-glucopyranosyl]-ceramides (**140**, **141**), 1-*O*-[α-l-fucopyranosyl-(1→11)-(*N*-glycolyl-α-d-neuraminosyl)-(2→4)-(*N*-acetyl-α-d-neuraminosyl)-(2→6)-β-d-glucopyranosyl]-ceramide (**144**), and homologous to each other 1-*O*-[(*N*-glycolyl-d-neuraminosyl)-(2→11)-(*N*-glycolyl-d-neuraminosyl)-(2→4)-(*N*-acetyl-d-neuraminosyl)-(2→6)-d-glucopyranosyl]-ceramides (**145**, **146**). The ceramide moieties of each compound were composed of sphingosine or phytosphingosine bases and 2-hydroxy- or nonhydroxylated fatty acid units (Figure 20). Gangliosides **134**, **139**–**146** demonstrated neuritogenic activity toward the rat pheochromocytoma cell line PC12 in the presence of NGF. The proportions of cells with neurites longer than the diameter of the cell body after the treatment with compounds **134**, **139**–**146** at concentration of 10 μM were of 40.2%, 50.8%, 34.0%, 35.7%, 39.1%, 43.0%, 43.0%, 40.2%, and 35.1%, respectively, in comparison with the control experiments (NGF, 5 ng/mL: 7.5%). The effects of **134**, **139**, and **142**–**145** were stronger than that of the mammalian ganglioside GM1 (35.6%). Compound **139** with an acetyl group at the terminal fucopyranosyl unit showed the most potent activity.

Enantiomeric pairs of sialic acids (d- and l-NeuAc) were converted to d- and l-arabinose, respectively, by chemical degradation [93]. Using this approach, the absolute configurations of the sialic acid residues NeuAc and NeuGc as d-forms were determined in the gangliosides from the sea cucumber *Cucumaria echinata*. Although naturally occurring sialic acids have been believed to have d-configurations on the basis of biosynthetic evidence, this is the first report describing the determination of the absolute configuration of the sialic acid residues in gangliosides using chemical methods.

Starfish and sea cucumbers gangliosides remain to be less studied, in comparison with cerebrosides. At the same time, about 30 new compounds and/or molecular species have been isolated since 2000. The carbohydrate chains of the starfish and sea cucumber gangliosides differ markedly from the carbohydrate chains of mammals as well as from each other. Generally, besides sialic acid residues, these compounds contain lactoside fragment (Gal-(1→4)-Glc-(1→1)-Cer) and analogous fragment additionally glycosylated with galactosamine (GalNAc-(1→3)-Gal-(1→4)-Glc-(1→1)-Cer). Part of them are derivatives of galactosylceramides having (Gal-(1→1)-Cer) moiety.

In the sea cucumbers gangliosides, containing fragments of only two cerebrosides were found: lactosides glycosylated with galactosamine (GalNAc-(1→3)-Gal-(1→4)-Glc-(1→1)-Cer), and glucosylceramides (Glc-(1→1)-Cer).

Both starfish and sea cucumber gangliosides contain unusual sialic acid residues, including sialic acids within carbohydrate chains as well as additional monosaccharide residues and unusual types of glycosidic bonds between them. For example, terminal β-d-Fuc*_f_* was found in the gangliosides from the starfish *Acanthaster planci*, 8-*O*-Me-NeuAc and 8-*O*-Me-NeuGc were found in the gangliosides from the starfish *Linckia laevigata* as well as the glycosidic bond 2→11 between sialic acid residues. The ganglioside from the starfish *Evasterias echinosoma* contains an unusual β-d-*N*-formyl-galactosamine residue, while the carbohydrate chains from gangliosides of the starfish *Patiria (=Asterina) pectinifera* bears the terminal α-L-arabinofuranose residue and has three forms of galactose (β-d-Gal_p_, β-d-Gal_f_, and α-d-Gal_p_). These gangliosides contain the maximum number of monosaccharide residues (up to nine), in comparison with other echinoderm gangliosides.

Gangliosides with the terminal α-l-Fuc*_p_* were identified in several species of sea cucumbers along with NeuAc and NeuGc residues within carbohydrate chains. A unique 8-*O*-sulfo-NeuAc residue was found in the corresponding substances from the sea cucumber *Stichopus chloronotus*. The maximum length of the carbohydrate chain in the sea cucumbers gangliosides was found in the ganglioside from *Stichopus japonicus*, which contained seven monosaccharide residues.

Lipid parts of gangliosides from both starfish and sea cucumbers were similar and contained both sphingosine and phytosphingosine bases of *normal*-, *iso*- and *anteiso*-types. Predominantly (2*R*)-2-hydroxy fatty acids of the normal type were found in these substances. For gangliosides of starfish and sea cucumbers, only one type of biological activity was studied, neuritogenic activity toward the rat pheochromocytoma cell line PC12 in the presence of NGF. In a number of cases, starfish and sea cucumbers gangliosides showed a higher neuritogenic effect at concentration 10 μM than the mammalian ganglioside GM1, while some gangliosides exhibited slighter action at the same concentration.

## 5. Conclusions

To the best of our knowledge, sphingolipids of 15 starfish and 9 sea cucumbers, mainly common Pacific Ocean inhabitants, have been studied (Table 1). In total, these 24 echinoderm species were used for the isolation and identification of about 150 sphingolipids. This indicates that echinoderms and, in particular, starfish and sea cucumbers are a rich source of sphingolipids, structures of which may differ markedly from the corresponding metabolites of plants and terrestrial animals.

Ceramides are the least studied group of echinoderms sphingolipids. Moreover, since 2000, only studies on starfish ceramides have been carried out. Nevertheless, a big variety of structural types of the isolated ceramides was detected, for instance, sphingosine and phytosphingosine LCBs of various lengths, *normal*-, *iso*-, and *anteiso*-types, often having one or two additional double bonds, were found in starfish ceramides. Fatty acid residues in starfish ceramides were most often identified as (2*R*)-2-hydroxy derivatives of various lengths (usually from C-18 to C-22) with normal hydrocarbon chains, which can also contain one additional double bonds. The “gray spot” in the study of starfish ceramides is the lack of data on biological activity, with the exception of the stimulating root growth of *Brassica campestris* activity by ceramides from *Asterias amurensis*.

Cerebrosides are the most studied class of starfish and sea cucumbers sphingolipids. Generally, about one hundred individual cerebrosides and their molecular species have been isolated from these animals. As in ceramides, sphingosine and phytosphingosine LCBs of various lengths with *normal*-, *iso*-, and *anteiso*-structures were found in starfish and sea cucumber cerebrosides. Unique oxidized sphingosine LCBs with additional hydroxy groups at either C-8 and C-9 or C-8 and C-11 were indicated in the sea cucumber *Stichopus japonicus*. Mainly saturated and monounsaturated (2*R*)-2-hydroxy fatty acids with normal hydrocarbon chains having various lengths were identified as constituents of these cerebrosides, but nonhydroxylated FAs were sometimes also detected. Almost all the isolated cerebrosides were monoglycosides and contained glucose or galactose residues. Cerebroside lactosides were isolated from the starfish *Luidia maculata*.

The following types of biological activities of starfish and sea cucumbers cerebrosides were studied: growth-promoting activity of *Brassica campestris*, anti-fatty liver activity in rats treated by orotic acid, alleviating adipose atrophy action in cancer-associated cachexia mice, effects on obesity-related metabolic disorders in mice, cytotoxic activities against KB, rat glioma C6 cells, and colon cancer Caco-2cells, and proangiogenic action. As result, it was shown that starfish and sea cucumbers cerebrosides possess various types of biological activities that are important for their practical application in the human diet and in the composition of food supplements (Table 1).

Starfish and sea cucumber gangliosides were also studied for some species, and their structural diversity was proved to be great. Carbohydrate chains of starfish and sea cucumbers gangliosides have interesting structural features and differ from gangliosides of terrestrial animals. Really, the residues of β-d-Fuc*_f_*, 8-*O*-Me-NeuAc, and 8-*O*-Me-NeuGc, β-d-*N*-formyl-galactosamine, as well as terminal α-l-Ara*_f_* were recently found in the starfish gangliosides. In gangliosides from holothurians (sea cucumbers), the terminal α-L-Fuc*_p_*, α-l-FucAc*_p_*, and 8-*O*-sulfo-NeuAc were detected.

For starfish and sea cucumbers gangliosides, only one type of biological activity was studied, namely, neuritogenic activity toward the rat pheochromocytoma cell line PC12 in the presence of NGF. Therefore, further research of other types of biological activities including antitumor and anti-inflammatory properties might be of interest. It is noteworthy that the starfish and sea cucumber gangliosides, as a rule, are species specific. Therefore, they could be taxonomic markers, such as some unusual starfish polar steroidal compounds [94,95] and sea cucumber triterpene glycosides [96]. However, the structures of gangliosides were less studied than those of other secondary metabolites of starfish and sea cucumbers and require further research.

Previously, we studied the metabolic profile of polar steroid compounds of three species of starfish and their changes under stress conditions, as well as the metabolic profile of triterpene glycosides from the sea cucumber *Eupentacta fraudatrix* [8,9,10,11,12,13]. The study of the metabolomic profiles of sphingolipids and their changes under various environmental conditions can also be one of the directions of metabolomics research. However, first of all, it is necessary to systematize the literature data on the structures of all types of sphingolipids, including ceramides, cerebrosides, and gangliosides, in these animals. We believe this review can help meet this challenge.

## Figures and Tables

**Table 1:** Composition and biological activity of starfish and sea cucumber sphingolipids mentioned in this review.

Order	Family	Scientific Name	Compounds			Type of Biological Activity	Ref.
			Ceramides	Cerebrosides	Gangliosides		
Class Asteroidea							
Forcipulatida	Asteriidae	*Allostichaster inaequalis*		**18**, **19**			[39]
		*Anasterias minuta*		**26**, **27**			[43]
		*Asterias amurensis*	**2**	**30**, **59**–**64**		Stimulating root growth *Brassica campestris* (**2**, **30**);	[23,50]
		*Distolasterias nipon*	**1**–**11**	**65**–**68**			[33,34]
		*Evasterias echinosoma*			**117**, **118**		[66]
		*Evasterias retifera*			**125**, **126**		[67]
		*Cosmasterias lurida*		**27**			[40]
Paxillosida	Luidiidae	*Luidia maculata*	**12**–**17**	**20**–**25**	**122**–**124**	Neuritogenic activity toward the rat pheochromocytoma PC12 cells in the presence of NGF (**124**)	[35,41,42,68,69]
Valvatida	Acanthaster-idae	*Acanthaster planci*			**115**, **116**	Binding epitope of AG2 pentasaccharide to human Siglec-2	[70,71,72,73]
	Asterinidae	*Patiria (=Asterina) pectinifera*			**127**	Neuritogenic activity toward the rat pheochromocytoma PC12 cells in the presence of NGF	[74]
	Ophidiasteridae	*Linckia laevigata*		**28**	**119**–**121**	Neuritogenic activity toward the rat pheochromocytoma PC12 cells in the presence of NGF (**120**)	[44,75,76,77]
	Oreasteridae	*Culcita novaeguineae*		**29**			[45]
		*Protoreaster nodosus*		**43**–**58**	**128**–**130**		[49,78]
		*Oreaster reticulatus*		**31**–**39**		(1) Mildly cytotoxic activity on the rat glioma C6 cells (**31**–**39**); (2) exertion of proangiogenic activity and increase of VEGF-induced human endothelial cell proliferation (**39**).	[46]
		*Narcissia canariensis*		**40**–**42**		Cytotoxic activity against KB cells (**40**)	[47]
Class Holothuroidea							
Holothuriida	Holothuri-idae	*Holothuria coronopertusa*		**69**–**86**			[52]
		*Holothuria pervicax*		**87**–**96**	**131**	Neuritogenic activity toward the rat pheochromocytoma PC12 cell line (**131**)	[53,79,80]
		*Holothuria leucospilota*		**102**–**105**	**132**–**134**	Neuritogenic activity toward the rat pheochromocytoma PC12 cell line (**132**–**134**)	[56,81]
		*Bohadschia argus*		**106**, **107**			[58]
Synallactida	Stichopodi-dae	*Stichopus japonicus*		**97**–**101**	**135**	(1) Absorption of cerebrosides in vivo and improving skin barrier functions (**97**–**101**); (2) neuritogenic activity toward the rat pheochromocytoma PC12 cells in the presence of NGF (**135**).	[54,55,82,83]
		*Stichopus chloronotus*			**136**–**138**	Neuritogenic activity toward the rat pheochromocytoma PC12 cells in the presence of NGF	[84]
Molpadida	Caudinidae	*Acaudina molpadioides*		**108**		(1) Anti-fatty liver activity of 108 in the rats with fatty liver induced by orotic acid; (2) alleviating adipose atrophy in the cancer-associated cachexia mice; (3) effects of cerebrosides on the obesity-related metabolic disorders in mice.	[59,61,62]
Dendrochi-rotida	Cucu-mariidae	*Cucumaria frondosa*		**109**–**114**		(1) In vitro cytotoxic activity against Caco-2 colon cancer cells (**112**–**114**); (2) in vitro antiadipogenic activity of cerebrosides.	[63,64,65]
		*Cucumaria echinata*			**139**–**141**, **144**–**146** **142**, **143**	Neuritogenic activity toward the rat pheochromocytoma PC12 cells in the presence of NGF	[85,86]
